# The Association of Residential Altitude on the Molecular Profile and Survival of Melanoma: Results of an Interreg Study

**DOI:** 10.3390/cancers12102796

**Published:** 2020-09-29

**Authors:** Eleonora De Martino, Davide Brunetti, Vincenzo Canzonieri, Claudio Conforti, Klaus Eisendle, Guido Mazzoleni, Carla Nobile, Federica Rao, Johannes Zschocke, Emina Jukic, Wolfram Jaschke, Georg Weinlich, Bernhard Zelger, Matthias Schmuth, Giorgio Stanta, Fabrizio Zanconati, Iris Zalaudek, Serena Bonin

**Affiliations:** 1 DSM-Department of Medical Sciences, University of Trieste, 34149 Trieste, Italy; edemartino@units.it (E.D.M.); brunetti.dav@gmail.com (D.B.); vcanzonieri@cro.it (V.C.); claudioconforti@yahoo.com (C.C.); stanta@impactsnetwork.eu (G.S.); fabrizio.zanconati@asugi.sanita.fvg.it (F.Z.); izalaudek@units.it (I.Z.); 2 Pathology Unit, IRCCS CRO Aviano-National Cancer Institute, 33081 Aviano, Italy; frao@cro.it; 3 ASU GI-Azienda sanitaria universitaria Giuliano Isontina, 34128 Trieste, Italy; 4 Azienda Sanitaria dell’Alto Adige, 39100 Bolzano, Italy; klaus.eisendle@sabes.it (K.E.); guido.mazzoleni@sabes.it (G.M.); carla.nobile@sabes.it (C.N.); 5 Institute for Human Genetics, Medical University of Innsbruck, 6020 Innsbruck, Austria; johannes.zschocke@i-med.ac.at (J.Z.); Emina.Jukic@i-med.ac.at (E.J.); 6 Department of Dermatology, Venereology and Allergology, Medical University of Innsbruck, 6020 Innsbruck, Austria; Wolfram.Jaschke@i-med.ac.at (W.J.); Georg.Weinlich@i-med.ac.at (G.W.); bernhard.zelger@i-med.ac.at (B.Z.); Matthias.Schmuth@i-med.ac.at (M.S.)

**Keywords:** cutaneous melanoma, altitude, miRNA, molecular profiling

## Abstract

**Simple Summary:**

Environmental factors such as UVR exposure and altitude of residence can contribute to the development of cutaneous melanoma. We hereby report that altitude of residence significantly associates with the molecular profiling of CM and melanoma specific survival. The fact that different miRNAs and transcriptomic profile vary with different geographical areas and residences altitude could support for possible regulatory mechanisms induced by environmental conditions, such as hypoxic environment and/or higher UVR exposure.

**Abstract:**

Cutaneous melanoma (CM) incidence is rising worldwide and is the primary cause of death from skin disease in the Western world. Personal risk factors linked to environmental ultraviolet radiation (UVR) are well-known etiological factors contributing to its development. Nevertheless, UVR can contribute to the development of CM in different patterns and to varying degrees. The present study aimed at investigating whether altitude of residence can contribute to the development of specific types of CM and/or influence its progression. To this aim, 306 formalin-fixed and paraffin-embedded (FFPE) tissues from primary CM diagnosed in different geographical areas were submitted to B-RAF proto-oncogene serine/threonine kinase (BRAF) and N-RAS proto-oncogene GTPase (NRAS) mutational status detection and mRNA and miRNA profiling by qPCR. Genes were chosen for their functions in specific processes, such as immune response (*CD2*, *PDL1*, or *CD274*) and pigmentation (*MITF*, *TYRP1*, and *TRPM1*). Furthermore, four microRNAs, namely *miR-150-5p*, *miR-155-5p*, *miR-204-5p*, and *miR-211-5p*, were included in the profiling. Our results highlight differences in the gene expression profile of primary CM with respect to the geographical area and the altitude of residence. Melanoma-specific survival was influenced by the gene expression of mRNA and miRNAs and varied with the altitude of patients’ residence. In detail, *TYRP1* and *miR-204-5p* were highly expressed in patients living at higher altitudes, unlike *miR-150-5p, miR-155-5p*, and *miR-211-5p*. Since miRNAs are highly regulated by reactive oxygen species, it is possible that different regulatory mechanisms characterize CMs at different altitudes due to the different environment and UVR intensity.

## 1. Introduction

Cutaneous melanoma (CM) incidence is rising worldwide and continues to be the primary cause of death from skin disease in the Western world [1]. Personal risk factors, including family history, multiple moles, fair skin, blue eyes, red hair, and freckles, together with environmental UV exposure are well-known etiological factors contributing to the development of CM [2,3]. Nevertheless, CM is not a homogeneous disease; there are different types of CM and UV radiation (UVR) can contribute to the development of melanoma in different ways and to varying degrees [2]. The same UVR that reaches the Earth’s surface can vary according to different variables. Well-known factors influencing UVR emissions are the ozone layer, the time of the day, the period of the year, and the latitude, with increasing UVR emission closer to the equator. Altitude is a further contributing factor, with an emission increment of 10−12% for every 1000 m elevation [4].

CM has been molecularly classified both at the genomic and at the transcriptomic levels [5]. At the genomic level, three subtypes have been identified: the *BRAF* subtype, the most common one, characterized by V600 amino acid residue mutations and the RAS subtype identified by the presence of *RAS* hot-spot mutations (*N-*, *K-*, *H-RAS*). The most frequent RAS mutations involve the *N-RAS*, especially the Q61 amino acid residue. Usually, *BRAF* and *NRAS* mutations are mutually exclusive [6]. The third group was defined by *NF1* mutations, which downregulate the RAS function through GTPase activity. A fourth group has been identified by the lack of hot-spot mutations in the above-mentioned genes; therefore, it has been named *Triple Wild-Type* [5]. At the transcriptomic level, three clusters of CM were recognized: the “immune” cluster, which includes CMs with high expression of immune cell-associated genes, immune signaling molecules, and receptors. The “keratin” subtype, which is the main representative group in primary melanomas, is characterized by high expression of genes associated with keratins, pigmentation, epithelium, and neuronal development. On the contrary, the last cluster called “MITF-low” is characterized by low expression of genes involved in pigmentation, epithelial, and several *MITF* gene targets [5]. Among all, the “keratin” cluster subtype appears to be worst one in terms of prognosis, while the “immune” group has been linked to improved survival [5].

Additionally, at the molecular level, microRNAs have been shown to be related to clinical variables, including CM outcome [7,8,9,10,11].

The objective of the present study was to investigate whether environmental variables, such as the altitude of residence, could contribute to the development of specific types of cutaneous melanomas, could promote it, and whether they can be linked to CM progression and/or survival.

To this aim, we selected primary CM cases of neighboring geographical regions that differed with respect to residential altitudes. In particular, we selected CM cases from patients living both at sea level (Trieste) and in mountainous areas (North and South Tyrol) and characterized these cases at the molecular level. With respect to patients living at sea level, we recruited residents of the province of Trieste with a diagnosis of cutaneous melanoma from both the University Hospital of Trieste and the National Cancer Institute of Aviano. Residents of the province of Bolzano (Bozen), Innsbruck, and Innsbruck-Land made up the group of mountainous area patients. Transcriptomic classification of the immune cluster was achieved by the quantification of *CD2* and *PDL1* mRNAs, while for keratin and MITF low clusters, the expression level of three pigmentation genes was analyzed, namely *MITF*, *TYRP1*, and *TRPM1* because of their different expression in “keratin” and “MITF-low” groups [5]. Furthermore, four microRNAs were also investigated for their possible association with clinical features, prognosis and survival [7,8,11,12,13,14,15], and transcriptomic analyses. In detail, miR-150-5p was linked with the tumor-infiltrated lymphocytes (TILSs) and with the proto-oncogene MYB, which plays a role in the regulation of hematopoiesis and tumorigenesis [8,11,15]. miR-155-5p, as a typical multifunctional microRNA, seems to modulate the immune response and, on the other hand, it interplays with miR-16 in TYRP1 regulation [16]. Consequently, miR-150-5p and miR-155-5p are physiologically involved in immunological processes. Both were reported to be potential biomarkers and targets for immunotherapies. The other two microRNAs, miR-204-5p and miR-211-5p, belong to the same family of miRNA (miR204/211 family) and act as tumor suppressors. They have already been linked with cancer features and tumor progression [7,12,13,14]. In addition, miR-211-5p acts as a metabolic switch, sensitizing melanocyte to oxygen deprivation, a lost mechanism in melanoma cells [17].

## 2. Results

The International Classification of Disease of Oncology (ICD-O) was applied to define the anatomical sites and the histopathological CM subtypes. In our cohort, only nodular, superficial spreading, and acral subtypes were numerically well represented; therefore, amelanotic, lentigo maligna, desmoplastic, spindle cell blue nevus-like, and epithelioid cell melanoma were merged in one group named “others”.

The cohort was subdivided into 3 different geographical areas of residence, namely the area of South Tyrol (BZ), the province of Trieste (TS), and the city of Innsbruck and Innsbruck-Land district (I/IL). Patients diagnosed at the National Cancer Institute of Aviano (CRO- Italy) were assigned to the group of Trieste (TS) residents.

### 2.1. Patients’ Features

Overall, 306 patients with a diagnosis of CM were included in the study: 107 (35%) cases were recruited from the Central Hospital of Bozen (Italy); 94 (31%) from the University Hospital of Trieste (Italy), 74 (24%) patients from the Medical University of Innsbruck (MUI-Austria), and 31 (10%) from the National Cancer Institute of Aviano (CRO-Italy).

Patients’ details are reported in Table 1. Women and men were equally represented (Chi-square test, *p* = 0.9), and there was no difference in gender distribution concerning altitude of residence (*p* = 0.5), tumor stage (*p* = 0.7), presence of lymph nodes (*p* = 0.5), Breslow’s thickness (*p* = 0.1), ulceration (*p* = 0.3), and histotype (*p* = 0.5). With respect to age, women were significantly older than men as shown in Figure S1 (*p* = 0.03).

Average patients’ age was 69 years (range 16–101 years) and average Breslow’s thickness was 4.6 mm (range 1.8−30 mm). Among the three cohorts, there was no significant difference between tumor features, even if stage II tumor was more represented in our dataset (*p* = 0.003) and nodular melanoma was the most frequent histotype in the group of TS and BZ (*p* < 0.0001 Table 1).

### 2.2. Mutational Characterization

Sample slides were tested for BRAF V600E and for NRAS Q61R mutations by immunohistochemistry. Samples with positive or doubtful staining were subjected to ddPCR analysis. Eighteen specimens were excluded from the mutation characterization because of lack of material. The resulting 288 of the 306 specimens were analyzed for BRAF and NRAS mutations: 96 (33.3%) patients were BRAF mutated (V600E or V600K), while 62 (21.5%) patients had mutations in the NRAS gene (Q61R, Q61K, Q61L, or Q61H). One sample with double positivity at the IHC was excluded from further analyses. All results are reported in Table 2.

Associations of the mutational status with patients’ CM features are listed in Table 3. Hereafter, only statistically significant results are reported. BRAF mutations were more frequent in men (*p* = 0.04), in CM located on the trunk (*p* = 0.02) and in younger patients (*p* = 0.0001). At the time of CM diagnosis, patients with BRAF V600 mutations were younger than wild type and NRAS Q61-mutated patients, (*p* = 0.0005 and *p* = 0.0001, respectively). In detail, age at diagnosis differed significantly with respect to the mutation burden: the mean age of BRAF-mutated patients was 62 years, which was lower than wild type (average age 71 years) and NRAS-mutated (average age 73 years) patients (*p* = 0.0001) (Table 3). 

In our cohort, the rate of BRAF-mutated CM in patients with positive LN involvement at diagnosis was significantly different in comparison with patients who did not have LN involvement (*p* = 0.02). The percentage of patients with positive lymph nodes was higher in BRAF-mutated patients (*n* = 25 of 96, 26%) than in wild type ones (*n* = 16 of 164, 12.9%). On the contrary, among LN-negative CMs, 81.5% of patients (*n* = 101 of 124) were wild type and 74% (*n* = 71 of 96) were BRAF mutated. In the primary logistic regression model (*n* = 288), age (years), sex, geographical area, altitude of residence, anatomical site of the CM, Breslow’s depth, presence of ulceration, and lymph node involvement were covariates investigated to affect the mutational status. Anatomical site (*p* = 0.011) and lymph node involvement (*p* = 0.049) had a statistically significant influence on the detected BRAF and NRAS mutation rate (overall regression *p* = 0.04- post estimation by Pearson χ2 goodness-of-fit test *p* = 0.3). The same analysis was repeated for the BRAF and NRAS mutations separately. BRAF mutation burden (*p* = 0.001- post estimation by Pearson χ2 goodness-of-fit test *p* = 0.4) depended significantly on age at diagnosis (*p* = 0.001) and in borderline significance on the anatomical site where CM developed (*p* = 0.05). For NRAS mutations, the regression results were not statistically significant (*p* =0.5).

### 2.3. Molecular Results and Geographic Features

Molecular results were compared with the environmental characteristics, namely the patients’ area of residence, which was subdivided into three groups, as described above, and the altitude of residence. Patients’ residence altitudes were significantly different between geographical areas (*p* < 0.0001), with a mean altitude of 53 m for Trieste (TS), 884 m for South Tyrol (BZ), and 699 m for Innsbruck and Innsbruck-Land (I/IL) (Table 1). Stage at diagnosis was different between geographical areas (*p* = 0.003, Table 1), because I/IL provided a minor number of cases due to the absence of informed consent from patients. By comparing stages between TS and BZ, no differences were found in CM stage distribution (*p* = 0.3).

**Figure 1:**
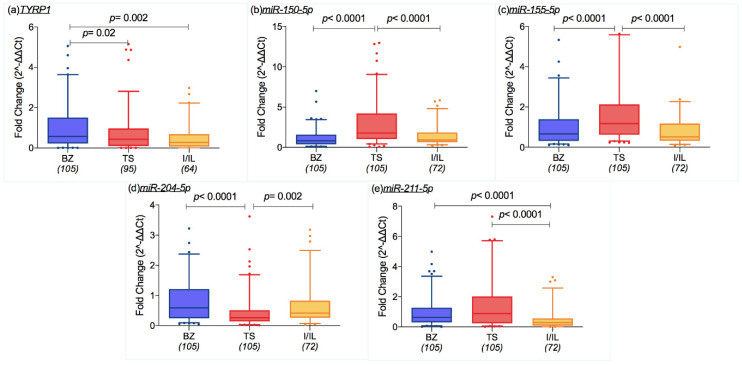
Box plots representing mRNA and miRNA expression levels (ratio of threshold cycles) that were different among the geographical areas: (**a**) Tyrosinase related protein 1 (TYRP1) was more expressed in the cohort of South Tyrol/Bozen (BZ), than in the province of Trieste TS and in the city of Innsbruck and Innsbruck-Land district (I/IL) (*p* = 0.02 and *p* = 0.002); (**b**) miR-150-5p and (**c**) miR-155-5p were more expressed in TS samples than BZ and I/IL ones (each comparison showed *p* < 0.0001); (**d**) miR-204-5p expression is lower in TS patients in comparison to those who live in BZ and I/IL (*p* < 0.0001, *p* = 0.002, in order); (**e**) miR-211-5p expression was lower in I/IL than the TS and BZ groups (both *p* < 0.0001). The box extends from the 25th to the 75th percentiles, the middle line indicates the median and the whiskers are the 5th and 95th percentiles (Mann–Whitney test).

*TYRP1* expression was higher in patients living in South Tyrol than in those living in TS and I/IL (Figure 1a, *p* = 0.02 and *p* = 0.002, respectively). Higher expression levels of *miR-150-5p* and *miR-155-5p* were recorded in patients living in TS in comparison to those living in BZ and I/IL (*p* < 0.0001 for both, Figure 1b,c). On the other hand, *miR-204-5p* (Figure 1d) was less expressed in patients living in TS than in those residing in BZ (*p < 0.0001*) and I/IL (*p* = 0.002). The expression of *miR-211-5p* was similar between patients living in Trieste and South Tyrol but differed between TS and I/IL residents (*p* < 0.0001, Figure 1e) and between BZ and I/IL residents (*p* < 0.0001) (Figure 1e).

**Figure 2:**
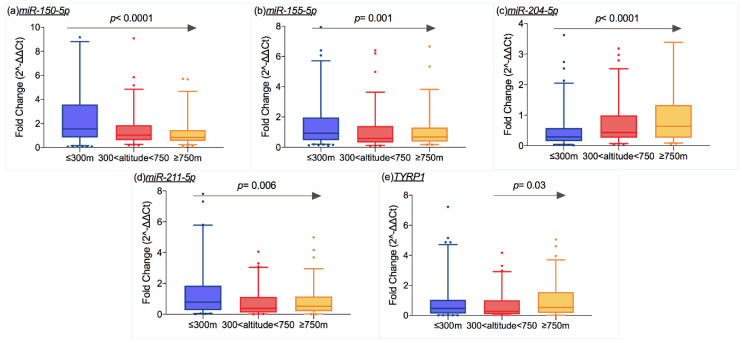
Box plots representing mRNA and miRNA expression levels (ratio of threshold cycles) that were different according to the altitude of residence: the expression of (**a**) miR-150-5p (*p* < 0.0001), (**b**) miR-155-5p (0.001), (**d**) miR-211-5p (*p* = 0.006) decreased as altitude increased, while the opposite trend was shown by (**c**) miR-204-5p (*p* < 0.0001). (**e**) TYRP1 expression increase from 300 m of altitude (*p* = 0.03). The box extends from the 25th to the 75th percentiles, the middle line indicates the median and the whiskers are the 5th and 95th percentiles (*p* values on the graph refer to Spearman’s correlation test). The altitude cut-off are ≤300 m, between 300 and 750 m, and ≥750 m.

As shown in Figure 2a,b,d, the expression of *miR-150-5p* (*p* < 0.0001, r = −0.3), *miR-155-5p* (*p* = 0.001, r = −0.2), and *miR-211-5p* (*p* = 0.006, r = −0.2) decreased in patients living at high altitude, while *miR-204-5p* expression was positively correlated with altitude of residence (*p* < 0.0001, r = 0.3) (Figure. 2c). The *TYRP1* expression increased starting from an altitude of 300 m above sea level (a.s.l.) (*p* = 0.03, r = 0.2) (Figure 2e).

### 2.4. Transcriptomic Profile and Demography

mRNA and microRNA expression levels were related to patients’ gender and age at the time of diagnosis. Hereafter, only statistically significant results are reported.

Based on a proposed cut-off at 75 years for elderly patients [18], data were grouped accordingly. Differences in CM staging and groupings were found (Chi-square test *p* = 0.01), namely the patients at stage II were older than stage I and III patients (Mann–Whitney test, *p* = 0.002 and *p* = 0.02, respectively). Furthermore, patients with positive lymph nodes were slightly younger than those with negative ones (Mann–Whitney test, *p* = 0.04).

The expression of *CD2*, *TRPM1*, and *miR-204-5p* decreased in patients aged equal or over 75 years, as shown in Figure S2 (*p* = 0.002, *p* = 0.001 and *p* = 0.04).

### 2.5. Transcriptomic Profile and CM Features

The expression levels of the analyzed mRNAs and miRNAs were related to CM characteristics, such as anatomical sites, Breslow’s thickness, presence of ulceration, and histotype.

Anatomical sites were defined according to the International Classification of Disease for Oncology (ICD-O) and divided into head&neck&face, trunk, upper limbs, and lower limbs. *TRPM1* expression level was lower in melanomas developed in the lower limbs than in those in the upper limb (*p* = 0.008), as shown in Figure S3. Furthermore, *miR-150-5p* showed a significantly higher expression in CM in the upper limbs compared to those in the lower limbs (*p* = 0.02) (Figure S3).

**Figure 3:**
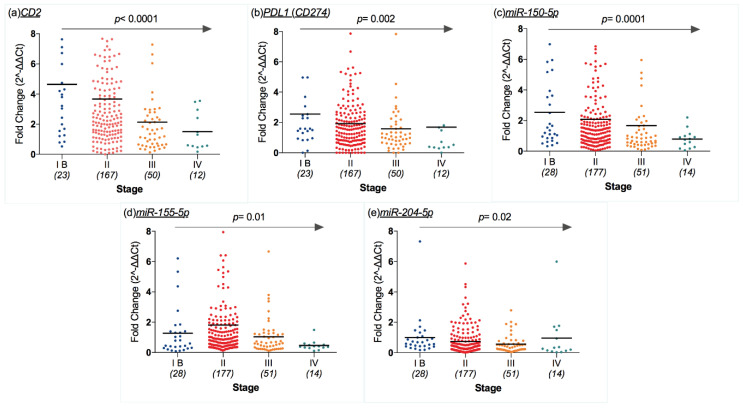
Scatter dot plots representing mRNA and miRNA expression levels (ratio of threshold cycles) that were different according to cutaneous melanoma’s (CM) stage: (**a**) CD2, (**b**) CD274, (**c**) miR-150-5p, (**d**) and miR-155-5p, and (**e**) miR-204-5p decreased as stage progressed (*p* < 0.0001, *p* = 0.002, *p* = 0.0001, *p* = 0.01 and *p* = 0.02). Middle lines refer to the median value (*p* values on the graph refer to Spearman’s rank correlation test using CM stage as the ranking variable).

In our cohort, *CD2*, *PDL1*, *miR-150-5p*, *miR-155-5p*, and *miR-204-5p* expression was significantly linked to CM stage, namely their expression decreases as stage progresses (*p* < 0.0001, *p* = 0.002, *p* = 0.0001, *p* = 0.01, and *p* = 0.02, respectively; Figure 3a–e).

AJCC staging parameters, such as the positivity of lymph nodes, tumor thickness, and ulceration, were significantly correlated to the expression levels of some mRNA and miRNA analyzed in this study. In particular, *CD2* and *PDL1* gene expression was lower in samples with positive lymph nodes (*p* = 0.0001 and *p* = 0.01, respectively; Figure S4). Similar results were obtained for *miR-150-5p* and *miR-155-5p* (Figure S4) (*p* = 0.0004 and *p* = 0.001, respectively).

**Figure 4:**
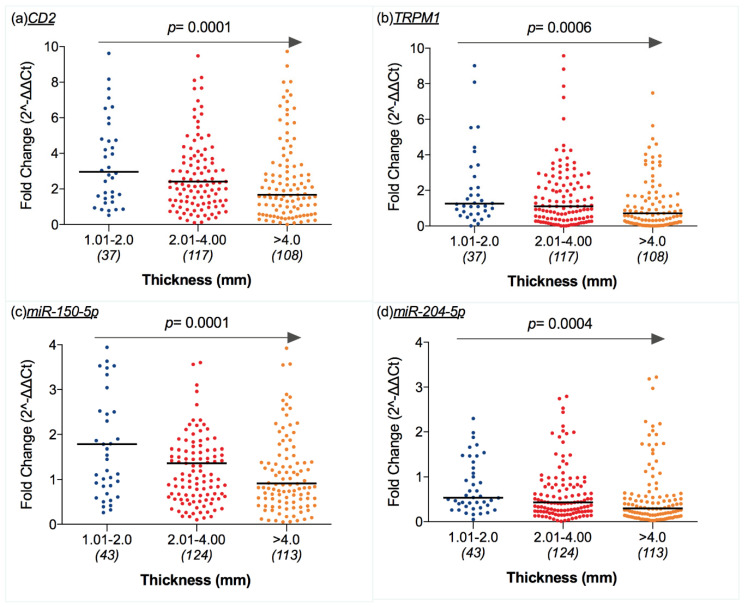
Scatter dot plots representing mRNA and miRNA expression levels (ratio of threshold cycles) were different according to CM thickness: (**a**) CD2 and (**b**) TRPM1 expression decreased as tumor thickness increased (*p* = 0.0001 and *p* = 0.0006); similarly, (**c**) miR-150-5p and (**d**) and miR-204-5p were negatively correlated with tumor thickness (*p* = 0.0001 and *p* = 0.0004). Middle lines refer to the median value (*p* values on the graph refer to Spearman’s rank correlation test using Breslow’s depth as a continuous variable).

Grouping samples according to T categories of CM [19] showed that expression levels of *CD2* and *TRPM1* decreased as CM thickness increased (*p* = 0.0001 and *p* = 0.0006, respectively; Figure 4a,b). Similar results were found for *miR-150-5p* and *miR-204-5p*, which were inversely correlated with CM thickness (*p* = 0.0001 and *p* = 0.0004, respectively; Figure 4c,d).

Regarding ulceration, expression levels of *TRPM1*, *MITF*, and *miR-204-5p* in our cohort were higher in non-ulcerated than ulcerated CMs (*p =* 0.006, *p* = 0.03, and *p* = 0.001, respectively).

The expression levels of the analyzed mRNAs and miRNA were related to the mutational status of CM. Accordingly, *CD2* expression levels were significantly higher in wild-type CM than in *NRAS*-mutated CM (*p* = 0.01). Considering both *BRAF* and *NRAS* mutations, the expression of *miR-150-5p* was higher in *BRAF*-mutated CM than in *NRAS*-mutated CM (*p* = 0.0001) (Figure S5).

### 2.6. Melanoma-Specific Survival and Overall Survival

Melanoma-specific survival (MSS) was significantly different among patients diagnosed in the different geographical areas (*p* = 0.0009), which may be related to the different distribution of CM stages reported above (Table 1). By stratifying survival data per CM stages, MSS was significantly different for stage II (*p* = 0.04) and stage IV (*p* = 0.01), whereby a shorter MSS was observed for residents from South Tyrol (BZ) and partially from Innsbruck and Innsbruck-Land (I/IL) (only for stage II CM).

**Figure 5:**
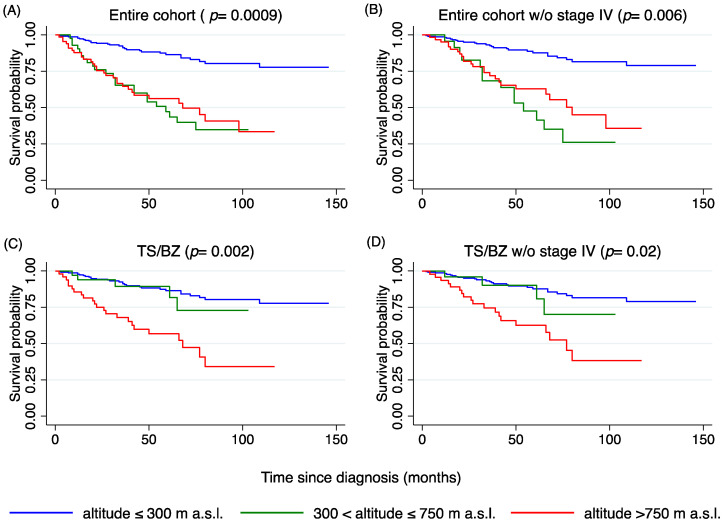
Kaplan Meier survival curves for melanoma-specific survival adjusted for age at diagnosis (MSS) for altitude of residence ranges (under 300 m, between 300 and 750 m, and above 750 m); (**A**) entire cohort, *p* = 0.0009; (**B**) entire cohort without stage IV CM, *p* = 0.006; (**C**) in cases from TS/BZ, *p* = 0.002 and (**D**) in cases from TS/BZ without stage IV CM, *p* = 0.02. The graphs of the survivor function were adjusted to the mean age of patients (57.6 years). MSS: melanoma-specific survival (*p* values on the graph refer to the log-rank test).

The residential altitude was divided into three groups as follows: (1) altitude of residence ≤300 m. a.s.l., (2), altitude of residence between 300 and 750 m, and (3) altitude of residence above 750 m a.s.l.. Considering the three groups, patients’ survival varied significantly with the residential altitude (*p* = 0.0009), with a shorter MSS for patients living above 300 m (Figure 5A). In order to exclude possible biases, stage IV CMs were excluded from survival analysis. The results confirmed that the altitude of residence influences the MSS (*p* = 0.006, Figure 5B). Since patients living in Innsbruck and Innsbruck-Land had a different stage distribution, survival analyses were restricted to patients living in the Trieste (TS) and Bozen region (BZ), where no differences were recorded with respect to stage distribution. Similar results were obtained both considering and excluding stage IV CMs, namely survival was shorter for patients living above 750 m a.s.l. (*p* = 0.002 and *p* = 0.02, respectively) as shown in Figure 5C,D.

Similar results were also found for overall survival (Figure S7), but the multivariate analysis did not return the altitude of residence as a variable associated to overall survival as shown in Table S4.

**Figure 6:**
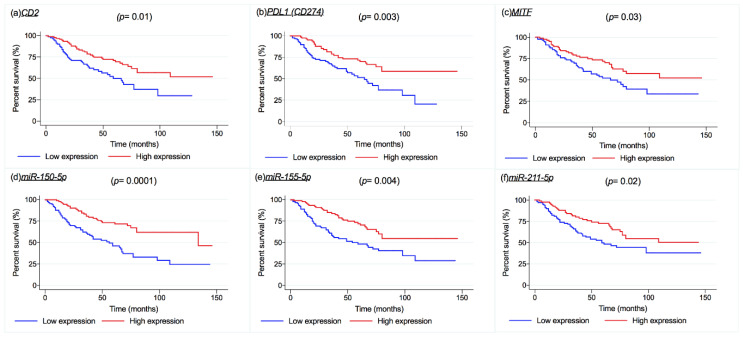
Kaplan Meier survival curves for melanoma-specific survival (MSS) for CD2, *p* = 0.01 (**a**); CD274, *p* = 0.003 (**b**); MITF, *p* = 0.03 (**c**); miR-150-5p, *p* = 0.0001 (**d**); miR-155-5p, *p* = 0.004 (**e**); and miR-211-5p, *p* = 0.02 (**f**). The gene and miRNA expression were dichotomized in low expression and high expression for the median value of each transcript (*p* values on the graph refer to the Log-rank test).

The influence of mRNA and miRNA on patients’ survival was investigated by dichotomizing mRNA and miRNA expression levels according to their median value. Over the entire cohort, melanoma-specific survival (MSS) was influenced by *CD2*, *PDL1* (CD274), and *MITF* (*p* = 0.01, *p* = 0.003, *p* = 0.03, respectively, Figure 6a–c). Higher expression levels of these transcripts were associated with longer MSS, showing a protective effect on survival. A similar result was found for *miR-150-5p*, *miR-155-5p*, and *miR-211-5p*, with higher expression levels associated with longer survival (in univariate analysis) as shown in Figure 6d,e,f (*p* = 0.0001, *p* = 0.004, and *p* = 0.02, respectively). However, multivariate Cox regression analysis did not confirm the dependence of MSS from the expression levels of those transcripts and miRNAs (Table 4). The regression also returned an effect of TYRP1 on patients’ MSS; in univariate analysis, the adverse effect of that gene was confined to stage II patients only (*p* = 0.04). In Relapse free survival analysis (RFS) only CD2 seemed to influence patients’ survival as shown in Figure S8.

## 3. Discussion

Our study adds new insights to the understanding of the complexity of intrinsic and environmental factors involved in melanoma development and progression. In particular, we observed that altitude of residence significantly correlates with the molecular profiling of CM and melanoma-specific survival. The expression levels of *miR-150-5p*, *miR-155-5p*, and *miR-211-5p* were significantly higher in patients living in Trieste (TS), which is a city located by the sea, compared to the geographic areas of South Tyrol (BZ) and Innsbruck/IL (I/IL), which are characterized by higher altitudes of residence (in our dataset, the mean altitude of the South Tyrol region is 884 m a.s.l. and of I/IL is 700 m a.s.l.). The opposite trend was found for *miR-204-5p* and *TYRP1*, which were significantly highly expressed in CM of patients living in South Tyrol. The study results were confirmed also when expression levels were correlated with altitude (a.s.l.). Actually, *miR-150-5p, miR-155-5p*, and *miR-211-5p* expression decreases as altitude increases; conversely, *miR-204-5p* and *TYRP1* were significantly higher in patients living at altitudes of or above 750 m a.s.l. A possible explanation for our finding might be related to an increased UVR and hypoxic environment at higher altitudes [20]. Concerning the *TYRP1* gene, it is well-known that the transcription factor Usf-1 activates the tyrosinase promoter by UV exposure [21]. Additionally, miRNA profiles have been shown to vary in sun-exposed skin [22] and that oxidative stress [23] can induce or suppress miRNA expression. Thus, a higher altitude with a hypoxic environment and increased UV intensity may account for the detected differences in the miRNA expression in our cohort. Although a very high affinity was found by mirDIP (http://ophid.utoronto.ca/mirDIP/) between miR-155-5p and HIF1A, the hypoxia inducible factor 1, as well as between miR-204-5p and HIF1AN, the hypoxia inducible factor 1 subunit alpha inhibitor, experimental data on HIF1A expression in our cohort did not support this hypothesis (see Tables S5 and S6). Micro RNA-155-5p, which was highly expressed in patients living at lower altitudes and at sea level, did not contribute directly to a decrease of the transcriptional activity through binding to HIF1A mRNA, in disagreement with others [24]. In vivo experiments also demonstrated the relationship between UV exposure and the downregulation of *miR-155-5p* in the development of squamous cell carcinoma (SCC) [25,26]. Furthermore, in vitro studies have shown that *miR-150-5p* is downregulated by hypoxia in pancreatic cell lines [27]. Nevertheless, the above-mentioned miRNAs have been described as favorable biomarkers in skin melanomas or as tumor suppressor miRNAs [9,14,28,29]. The fact that different miRNAs have been detected in different geographical areas characterized by different altitudes supports the hypothesis for possible regulatory mechanisms induced by environmental conditions, such as a hypoxic environment and/or higher UVR exposure. So far, however, no evidence has been found in our data to support this hypothesis. We acknowledge anyway that the hypobaric hypoxia at altitudes ranging from 564 to 2153 m a.s.l. of patients living in Bozen, Innsbruck and its land can be negligible. 

Altitude of residence at or over 750 m a.s.l. negatively affected patients’ melanoma-specific survival; however, even though statistically significant, the revealed risk factor by Cox regression was 1. Even if this result may be biased by an unequal distribution of CM stages among the different geographic groups, it remained statistically significant when considering only the cases from South Tyrol (BZ) and Trieste (TS), which were equally distributed. In line with this finding, three suppressor miRNAs analyzed in this study (namely *miR-150-5p, miR-155-5p*, and *miR-211-5p*) were more expressed in residents at low altitude level. Although the amount of UVR increases with altitude, a direct causality between the altitude of residence and CM mortality appears unlikely. Even though populations living at high altitude are exposed to chronic hypobaric hypoxia and permanent stress due to hypoxic exposure, it seems that cancer mortality is reduced in populations living at higher altitude over a broad spectrum of cancer types [30]. Rather, other logistic and societal/cultural issues may play a role, such as difficulties encountered by patients living in rural areas to reach hospitals for treatment or poor knowledge about symptoms. Interestingly, an Austrian study revealed that CM incidence rates have been reported to increase with altitude, but mortality rates decrease [31]. Previously, higher melanoma prevalence was reported at higher altitude of residence, but no influence on melanoma specific mortality was documented [32]. The Cox regression has shown in our cohort that the diagnosis of a stage IV melanoma was the main variable affecting patients’ survival.

All the miRNAs analyzed in our study have proven to be significantly associated with favorable prognostic factors. In particular, *miR-150-5p* and *miR-155-5p* were found to be inversely associated with Breslow’s depth, stage, and lymph node involvement. Furthermore, they seem to positively influence melanoma-specific survival. In line with us, Tembe and colleagues associated *miR-150-5p* with longer survivors and inversely associated to melanoma stage (mainly III and IV) [33]. Our findings seem to be confirmed by the more recent overexpression of *miR-150-5p*, which was found to inhibit the proliferation, migration, and invasion of melanoma cells [15]. Possible mechanisms explaining its favorable activity in melanoma are the inhibition of the transcription factor MYB [15] and its association to aerobic glycolysis through SIX1 repression [28]. Regarding *miR-155-5p,* this was already identified among upregulated miRNAs, predicting good prognosis after surgical excision [11]. This miRNA seems to bind and translationally inhibit *TYRP1* mRNA [16]. Indeed, two competing miRNAs pair, namely *miR-16* acting as a stabilizer and *miR-155-5p* as an inhibitor, inter-play in the *TYRP1* stability [34].

The other two miRNAs studied here, namely *miR-204-5p* and *miR-211-5p,* belong to the same family (microRNA mir204/211 family) and they are encoded by the *TRPM3* and *TRPM1* gene, respectively. In our cohort, *miR-204-5p* was inversely associated with melanoma ulceration, stage, and depth. Similar results were highlighted by Galasso and co-workers on the association of *miR-204-5p* with important clinical parameters, such as Breslow’s depth [7]. Our results show that *miR-211-5p* favorably influenced melanoma-specific survival but did not influence the overall survival. In disagreement with us, Lu and colleagues found a negative influence of *miR-211-5p* on patients’ overall survival [10], even if the development of most melanomas has been specifically associated with the depletion of *miR-211* transcript levels, thus supporting its positive effect [17]. In agreement with our results, Santoni and co-workers reported that the *miR-211* expression is linked to tumor suppressor functions [29]. Furthermore, a direct connection has been shown between *miR-211-5p* and *MITF*, as it acts as a transcription factor for *TRPM1*, which within its sixth intron encodes for *miR211-5p* [17]. Consequently, *MITF* has been shown to suppress melanoma metastasis through its transcriptional activation of *miR-211* via the *TRPM1* promoter. In our cohort, similarly to *miR-211-5p*, *MITF* expression was also associated to longer melanoma-specific survival, findings that are supported by Mazar and colleagues [17]. Interestingly, *TRPM1* was not directly linked to patients’ survival but to favorable pathological variables, such as Breslow’s depth and absence of ulceration, which is in line with other authors who defined *TRPM1* loss as an excellent marker of melanoma aggressiveness [29,35,36]. Immune response molecules, namely *CD2* and *PD-L1*, were chosen in our study to define immune-type melanomas, which have been associated with improved patients’ survival [5]. Accordingly, in our samples, *CD2* and *PD-L1* were both associated to variables of good prognosis, such as lower CM stages, thinner CM, as well as to longer CM-specific survival. Consequently, *CD2* has already been proposed as an independent predictor of disease recurrence and overall survival among patients with primary cutaneous melanoma [37]. The fact that CMs have higher expression of *PD-L1* and *CD2* is most likely due to an anticancer immune response, as is usually indicated by tumor-infiltrating lymphocytes [37,38].

Our data suggest that the mutational status of BRAF-V600 and NRAS-Q61 did not vary among geographical regions and altitudes of residence. Regression analysis confirmed that mutational burden was an independent variable that was not influenced by altitude of residence but by some clinical characteristics of CM [39,40,41] and patients’ demographics. Contrary to previous studies [42,43], in our cohort, BRAFV600 mutations were more frequently detected in men. This result is likely due to our selection criteria as CMs of male patients were analyzed from the trunk, which has already been reported as a preferential anatomical site associated with BRAF mutation [44]. Patient age showed a clear influence on the documented BRAF mutation rate in this study, with younger patients prevailing in the BRAF-mutated group in comparison with NRAS-mutated as well as BRAF/NRAS wild-type ones. BRAF-mutated melanomas are indeed characterized by a young age at diagnosis, absence of chronic sun damage of the skin, and melanoma occurrence on the trunk [45], which closely reflect the features of our BRAF-mutated patients. NRAS mutations were frequently recorded in CMs located on the lower limbs (7.7%, *n* = 22 of 287) in our dataset, in agreement with others [44,46]. The trunk is commonly considered a site of intermittent sun exposure, while the lower limbs a chronic sun-damaged skin area (CSD). Therefore, our data confirm that the type of UV exposure is a determinant influencing the mutational burden of BRAF V600 and NRAS Q61 [44,46].

The overall detection rate of BRAF in our cohort was 33% (*n* = 96 of 288), which appears to be in line with the current literature [47]. *NRAS* mutation frequencies accounted for 21% (*n* = 62 of 288) in our study, which is also in line with the reported data [48]. Notably, NRAS-mutated melanomas in our study were related to lower *CD2* expression levels. The latter finding might be linked to oncogenic RAS, which can disrupt antitumor immunity, resulting in decreased immunogenicity of the RAS-transformed cells [49].

## 4. Materials and Methods

### 4.1. Patients and Samples

This was a population-based retrospective study. The study was part of the MEMS project (www.mems-interreg.eu) funded by the European Union’s Interreg V-A Italia-Austria 2014−2020 program. Participating centers included the University Hospitals of Trieste (Italy) and Innsbruck (Austria), the Central Hospital of Bolzano (Italy), and the National Cancer Institute of Aviano (CRO-Italy). The study was conducted in accordance with the Declaration of Helsinki, and it was approved by the following Ethical Committees: Ethical Committee of the Health Agency of the province of Bozen (protocol number 0081449, 01/07/2019), Ethical Committee of the Friuli-Venezia Giulia region (protocol number 20817, 02.07.2018), and the Ethical Committee of the Medical University of Innsbruck (protocol number 1177/2017 and 1182/2018). Written informed consent was obtained from all individual participants included in the study with the exception of deceased patients (permission granted by the Ethical Committee).

Cases of primary CM diagnosed between 01/01/2006 and 31/12/2015 included in this study were retrieved from the histopathological databases at the corresponding participating centers.

Inclusion criteria were the following: (i) Unequivocal diagnosis of primary cutaneous melanoma with a tumor thickness ≥ 1.8 mm (this thickness was chosen to provide tissue slides for molecular analyses without consuming the entire tissue block); (ii) location in the head/neck, trunk/limbs, and acral sites—trunk and head (neck and face) were chosen for men, and higher and lower limbs for women. These sites were chosen because they are mostly gender-related. Cases were matched among participant groups per gender, anatomical site, age range, and tumor thickness; and (iii) availability of the fixed- and paraffin-embedded block. The cohort included also patients with criteria (i), (ii), and (iii) of any anatomical site in patients aged ≥90 years or <30 years as extreme values of the sample. In patients with more than one primary CM, only the first CM was included while subsequent melanomas were excluded from the study.

All CMs were reviewed and grouped into stages according to the AJCC TNM classification 8th edition [19]. Moreover, for all cases, we recorded Breslow’s thickness, presence/absence of ulceration, and anatomical site. With regard to patients’ demographics, we recorded gender, age at diagnosis, and altitude of the geographical area of residence. In addition, follow-up data were collected from the date of diagnosis of the primary CM until 31/12/2018 or death.

The dataset was also analyzed for the altitude of residence.

### 4.2. Immunohistochemistry

Four or 2-μm-thick sections were cut from each tissue block for immunohistochemical staining using a fully automatized assay based on the BenchMark ULTRA system (Ventana-Roche Diagnostics, Tucson, AZ 85755, USA). The following antibodies were used: the VE1 clone (Ventana Medical System, Tucson, AZ 85755, USA) at a dilution of 1:100 for the detection of BRAF V600E mutation; the rabbit monoclonal anti-human N-Ras Q61R, clone SP174 (Ventana Medical System-Abcam, Tucson, AZ 85755, USA) at a dilution of 1:100 for NRAS Q61R analysis. Sections were dried at 60°C for 30 min and after dewaxing, the slides were pretreated with Cell Conditioning 1 (Ventana Medical System, Tucson, AZ 85755, USA) for 64 min for antigen unmasking and submitted to peroxidase inhibition. Primary antibody decoration was carried out at 37 °C for 16 min for BRAFV600E and 32 min for NRASQ61R. Chromogenic detection was performed using Diaminobenzidine chromogen (DAB) for BRAFV600E, while for NRASQ61R using the UltraView Universal Alkaline Phosphatase Red Detection Kit (Ventana Medical System, Tucson, AZ 85755, USA). Counterstaining was performed with Hematoxylin II for 5 min. After washing, slides were mounted as usual.

Concerning the immunohistochemistry analyses, tissue sections were scored as positive when at least 10% of melanoma cells were positively stained.

The criteria for unequivocal positive BRAF and NRAS staining included strong and medium cytoplasmic staining. Weak cytoplasmic diffuse staining and no staining were scored as negative. Tissue sections with equivocal staining were checked through digital droplet PCR (ddPCR) to confirm the presence or the absence of BRAF/NRAS mutations.

### 4.3. DNA Extraction and Digital Droplet PCR (ddPCR)

DNA was extracted from five 10-µm-thick tissue sections using the Maxwell^®^ RSC (Promega, Madison, WI 53711-5399, USA) device according to the manufacturer’s instructions. Samples were stored at 4 °C until use. BRAF and NRAS mutations were detected by digital droplet PCR (Biorad, Hercules, CA 94547, USA; Cat. No. 12001037, Cat. No. 12001006). Primers and probes for wild-type and mutated *BRAF* and *NRAS* genes were purchased by Biorad (Biorad, Hercules, CA 94547, USA; Cat. No. 12001037, Cat. No. 12001006). The reaction was carried out in a solution containing 10 μL of ddPCR^TM^ Supermix for probes (no dUTP) (Biorad, Hercules, CA 94547, USA; Cat. No. 1863024), 1 μL of mutations Screening Assay, and 25 ng of DNA. The droplets were generated using a QX200^TM^ Droplet Generator (BioRad, Hercules, CA 94547, USA). Subsequently, emulsions were transferred onto a 96-well PCR plate (Biorad, Hercules, CA 94547, USA) and submitted to PCR amplification as follows: denaturation for 10 min at 95 °C, 40 cycles of 94°C for 30 s, annealing/extension for 1 min at 55 °C, and one cycle at 98 °C for 10 min. After amplification, droplets were read in the QX200^TM^ Droplet Reader (BioRad, Hercules, CA 94547, USA). Data analysis was performed using QuantaSoft^TM^ software.

### 4.4. RNA Analysis

#### 4.4.1. Total RNA extraction

Five 10-μm-thick sections were cut from micro-dissected FFPE tissue blocks and total RNA was isolated using a Maxwell^®^ RSC (Promega, Madison, WI 53711-5399, USA) device according to the manufacturer’s instructions. The protocol was subdivided into two parts: the first part refers to the Maxwell^®^ RSC RNA FFPE kit (Cat. No. AS1440, Promega, Madison, WI 53711-5399, USA) where the sections were de-waxed, digested with proteinase K, de-crosslinked, and treated with DNase I. The subsequent steps follow the Maxwell^®^ RSC miRNA Tissue Kit instructions (Cat. No. AS1460, Promega, Madison, WI 53711-5399, USA). The 1-Thioglycerol/Homogenization solution and the Lysis Buffer (MC501C) were added to the aqueous phase. The entire volume of lysate was added to Maxwell^®^ RSC Cartridge and extracted with its specific method. The total RNA was eluted in 50 μL of nuclease-free water. Samples were split into aliquots and stored at −80 °C.

#### 4.4.2. Reverse Transcription and Real-Time PCR Assays of mRNAs

As regards the analysis of target mRNA gene expression, cDNA was synthesized using random primers and 250 U M-MLV reverse transcriptase (Thermo Fischer Scientific, Waltham, MA 02451, USA; Cat. No. 28025013) as previously described [50]. As regards quantitative Real-time PCR, 60 ng of cDNA was added to JumpStartTM 2X Taq ReadyMixTM (Sigma-Aldrich^®^, St. Louis, MO 63103, USA; Cat. No. D7440), together with 210 nM of TaqMan probe (IDT, Coralville, IA 52241, USA), 320 nM of reverse and forward primers (IDT, Coralville, IA 52241, USA), Rox100, and made up to a final volume of 21 μL with sterile water. Primers and Taqman probes were designed using the on-line Primer3Plus tool (see Table S1) [51] and their specificity was checked through the Genome Browser BLAT tool [52]. Each reaction was carried out in duplicate. Real-time PCR analysis was performed on a Mastercycler^®^ ep Realplex (Eppendorf, Hamburg, Germany) as reported in Table S1. For relative quantification, the “delta-delta Ct” method [53] was used where, as housekeeping, the geometric mean of at least 2 genes out of 4 analyzed in the present study was employed (see Supplementary Materials for details and Table S3 for efficiencies). Samples with a low concentration of total RNA and samples processed with Bouin’s fixative were excluded from the expression analysis.

#### 4.4.3. Reverse Transcription and Real-Time PCR Assays of MicroRNAs

For miRNA detection, 40 ng of total RNA were reverse transcribed using the miRCURY^®^ LNA^®^ RT kit (Qiagen, Hilden, Germany; Cat. No. 339340) according to the manufacturer’s protocol. Real-time PCR was carried out using 0.4 ng of diluted cDNA (40X), Fast EvaGreen^®^ qPCR Master Mix 2X (Biotium, Fremont, CA 94538, USA; Cat. No. 31003), and 1 μL of specific pre-designed validated miRCURY^®^ LNA^®^ miRNA PCR Assays (QIAGEN, Hilden, Germany; Cat. No. 339306) in a total reaction volume of 10 μL (see Tables S2 and S3 for details).

Each reaction was run in duplicate. Real-time PCR analysis was performed on a Mastercycler^®^ ep Realplex (Eppendorf, Hamburg, Germany) using the following cycles’ conditions: 95 °C for 10 min, 45 cycles of 95 °C for 10 s, and 60 °C for 1 min. Furthermore, at the end of the procedure, the melting curve analysis was performed to evaluate the specificity of the amplified products. For relative quantification, the delta-delta Ct method was applied [53].

### 4.5. Molecular Subtypes

Samples were clustered into transcriptomic subtypes based on their high or low expression level as described in the TCGA classification [5] (see Supplementary Materials for details and Figure S6 for results).

### 4.6. Statistical Analyses

Parametric or non-parametric tests were performed after checking data distribution (D’Agostino-Pearson omnibus normality test and Shapiro-Wilk normality test). For normally distributed variables, the *t*-test or ANOVA test was used. The association of the ratio of the investigated genes at the mRNA level and miRNA with categorical variables was analyzed with the Mann–Whitney test. The Spearman’s rank correlation test was run to identify a possible correlation between Breslow’s depth and mRNA or miRNA expression levels.

Trends across the ordered group were assessed by submitting mRNA and miRNA expression levels to an extension of the Wilcoxon rank-sum test developed by Cuzick [54]. For survival analysis, the normalized RT-qPCR ratios of the analyzed genes and miRNAs were dichotomized according to their median value. Patients with lower gene expression in comparison with the corresponding median value were classified as lower status, while patients with higher values were classified as higher status of gene expression. The log-rank test was used to investigate whether each molecular and clinical-pathological variable affected patients’ survival.

For melanoma-specific survival (MSS), the time between the date of diagnosis and the date of death or last follow-up was used; for overall survival (OS), the period of time from diagnosis to death from any cause or last follow-up was considered. The Cox proportional hazard regression method was applied to analyze patients’ demographics (age at diagnosis, gender, altitude of residence, geographical areas), pathological covariates (stage, ulceration, anatomical site), mutational status, and gene and miRNA expression levels in the entire cohort of patients, to test the joint effects of the covariates on patients’ survival. The proportional hazard assumption was checked by Shoenfeld’s residuals method.

The combined impact of multiple parameters on routine BRAFV600 and/or NRASQ61 mutational status was evaluated through logistic regression analysis. A statistical model to predict the probability of BRAF mutations for individual patients was developed, including age at diagnosis, sex, geographical area of residence, altitude of residence, Breslow’s depth, the presence of ulceration, and the lymph node involvement at diagnosis as independent variables. Pearson χ2 goodness-of-fit test was used to test for the fitted model.

Outliers were excluded from the box-whiskers plots and scatter-dot plot representations. All statistical analyses were two-sided and values of *p* < 0.05 were considered statistically significant. Statistical analysis was carried out with the GraphPad Prism 6.0 software (San Diego, CA 92108, USA) and the Stata/SE 16.0 package (StataCorp, College Station, TX 77845-4512, USA).

## 5. Conclusions

The MEMS project was a retrospective study where primary cutaneous melanomas were analyzed at the molecular level and these characteristics were related with pathological and environmental features. The heterogeneous cohort was composed of patients who had been living in different geographic areas and at different altitudes of residence.

In conclusion, our findings highlight that there are differences in the expression profiles of certain mRNAs and miRNAs with respect to the altitude of residence. Namely, *TYRP1* and *miR-204-5p* were highly expressed in patients living at higher altitudes, unlike *miR-150-5p, miR-155-5p*, and *miR-211-5p*. Since miRNAs are highly regulated by reactive oxygen species, it is possible that different regulatory mechanisms characterize CMs at different altitudes due to the different environment and UVR intensity.

According to our results, shorter melanoma-specific survival is recorded in patients living at higher altitudes, which is likely due to societal/cultural issues rather than to a causative effect of the residence at higher altitude. This finding could be associated to our molecular results; indeed, the expression of the onco-suppressor *miR-150-5p*, *miR-155-5p*, and *miR-211-5p* were negatively associated with the residence altitude.

Admittedly, our investigation on the effect of URV exposure has not taken into consideration the patients’ periods of residence and their recreational and work activities: This is an important limitation of the present study. Therefore, the results of our study on the survival and altitude of residence need to be confirmed by further studies. 

## Figures and Tables

**Table 1:** Dataset characteristics.

Dataset Characteristics						
Variable and its description		BZ *n* = 107 (35%)	TS *n* = 125 (41%)	I/IL *n* = 74 (24%)		Total *n* = 306 (100%)
Geographical characteristics						
Altitude of residence	Mean in meter (range)	884 (214–2153)	53 (0–253)	699 (564–1513)	*p* < 0.0001 ^†^	279 (0–2153)
Demographic characteristics						
Gender	Male	59 of 107 (55%)	66 of 125 (52.8%)	41 of 74 (55.4%)	*p* = 0.9 ^‡^	166 of 306 (54%)
	Female	48 of 107 (45%)	59 of 125 (47.2%)	33 of 74 (44.6%)		140 of 306 (46%)
Mean age	Year (range)	67 (19–101)	69 (16–98)	70 (21–100)	*p* = 0.5 ^†^	69 (16–101)
Pathological characteristics						
Localization ^§^	Head&Neck&face ^a^	25 of 107 (23.4%)	29 of 125 (23.2%)	18 of 74 (24.3%)	*p* = 0.9 ^‡^	72 of 306 (23.5%)
	Trunk ^b^	35 of 107 (32.7%)	41 of 125 (32.8%)	25 of 74 (33.8%)		101 of 306 (33%)
	Upper Limb ^c^	16 of 107 (15%)	16 of 125 (12.8%)	12 of 74 (16.2%)		44 of 306 (14.4%)
	Lower Limb ^d^	30 of 107 (28%)	35 of 125 (28%)	18 of 74 (24.3%)		83 of 306 (27.1%)
	Other sites ^e^	1 of 107 (0.9%)	4 of 125 (3.2%)	1 of 74 (1.4%)		6 of 306 (2%)
Stage	I B	10 of 107 (9.3%)	9 of 125 (7.2%)	12 of 74 (16.2%)	*p* = 0.003 ^‡^	31 of 306 (10%).
	II	68 of 107 (63.6%)	89 of 125 (71.2 %)	34 of 74 (45.9%)		191 of 306 (62%)
	III	21 of 107 (19.6%)	25 of 125 (20%)	9 of 74 (12.2%)		55 of 306 (18%)
	IV	6 of 107 (5.6%)	2 of 125 (1.6%)	9 of 74 (12.2%)		17 of 306 (6%)
	unknown	2 of 107 (1.9%)	0 of 125 (0%)	10 of 74 (13.5%)		12 of 306 (4%)
Lymph nodes	Positive	23 of 107 (21.5%)	22 of 125 (17.6%)	13 of 74 (17.6%)	*p* = 0.7 ^‡^	58 of 306 (19%)
	Negative	82 of 107 (76.6%)	103 of 125 (82.4%)	51 of 74 (68.9%)		236 of 306 (77%)
	unknown	2 of 107 (1.9%)	0 of 125 (0%)	10 of 74 (13.5%)		12 of 306 (4%)
Breslow’s thickness	Mean in mm (range)	4.5 (1.8−27.6)	4.6 (1.8−30)	4.6 (1.8−30)	*p* = 0.9 ^†^	4.6 mm (1.8−30)
Ulceration status	yes	52 of 107 (48.6%)	65 of 125 (52%)	23 of 74 (31%)	*p* = 0.3 ^‡^	140 of 306 (46%)
	no	54 of 107 (50.5%)	57 of 125 (45.6%)	34 of 74 (46%)		145 of 306 (47%)
	unknown	1 of 107 (0.9%)	3 of 125 (2.4%)	17 of 74 (23%)		21 of 306 (7%)
Histotype ^¶^	NOS ^f^	2 of 107 (1.9%)	46 of 125 (36.8%)	37 of 74 (50%)	*p* < 0.0001 ^‡^	85 of 306 (27.7%)
	Nodular ^g^	57 of 107 (53.3%)	61 of 125 (48.8%)	22 of 74 (29.7%)		140 of 306 (46%)
	Superficial spreading ^h^	32 of 107 (29.9%)	1 of 125 (0.8%)	7 of 74 (9.5%)		40 of 306 (13%)
	Acral ^i^	5 of 107 (4.7%)	0 of 125 (0%)	3 of 74 (4.1%)		8 of 306 (3%)
	Others ^l, m, n, o, *p*, q, r^	10 of 107 (9.3%)	17 of 125 (13.6%)	5 of 74 (6.8%)		32 of 306 (10%)
	Unknown	1 of 107 (0.9%)	0 of 125 (0%)	0 of 74 (0%)		1 of 306 (0.3%)

^†^ Ordinary one-way ANOVA test (the unknown cases were excluded from the analysis); ^‡^ Chi-square test (the unknown cases were excluded from the analysis); underlined *p* highlights statistically significant results by univariate analysis (*p* < 0.05). § ICD-O code was used to define anatomical tumor localization: ^a^. C44.0; C44.1; C44.2; C44.3; C44.4; ^b^. C44.5; ^c^. C44.6; ^d^. C44.7 and ^e^. C51.0; ^¶^ SNOMED code was used to define tumor histotypes: ^f^. not specified; ^g^. M87213; ^h^. M87433; ^i^. M87443; ^l^. M87453; ^m^. M87723; ^n^. M87733; ^o^. M87803; *^p^*. M87303; ^q^. M87423; ^r^. M87713.

**Table 2:** Mutational characteristics of the whole dataset.

Mutation Status	*n* = 288	(100%)
BRAF ^a^	*n* = 96	(33.3%)
NRAS ^b^	*n* = 62	(21.5%)
Focal positivity for NRAS ^b c^	*n* = 5	(1.8%)
BRAF and NRAS negative	*n* = 124	(43%)
Positive IHC for BRAF and NRAS	*n* = 1	(0.4%)

^a^: BRAF mutations V600E or V600K; ^b^: NRAS mutations Q61R or K or L or H; ^c^: one sample is defined with focal positivity (−/+) by IHC and four both through IHC and ddPCR (0.08 or 0.16 copies/μL).

**Table 3:** Mutational status and patients’ cutaneous melanoma features. Underlined *p* highlights statistically significant results (*p* < 0.05).

Characteristics		BRAF Mutated	NRAS Mutated	No Mutated	Total	*p* Value	*p* Value BRAF Mut vs. No Mutated	*p* Value NRAS Mut vs. No Mutated	*p* Value BRAF Mut vs. NRAS Mut
		*n* = 96 (33.5%)	*n* = 67 (23.3%)	*n* = 124 (43.2%)	*n* = 287 (100%)				
Environmental characteristics									
Geographic areas	BZ	33 of 96 (34.4%)	30 of 67 (44.8%)	43 of 124 (34.7%)	106 of 287 (37%)	0.2 ^‡^	0.2 ^‡^	0.3 ^‡^	0.4 ^‡^
	TS	45 of 96 (46.9%)	24 of 67 (35.8%)	46 of 124 (37.1%)	115 of 287 (40%)				
	I/IL	18 of 96 (18.8%)	13 of 67 (19.4%)	35 of 124 (28.2%)	66 of 287 (23%)				
Altitude of residence	Mean (range)	449.4 (13–1718)	593 (46–2153)	509 (10–2131)	660 (10–2153)	0.1 ^†^	0.4 ^††^	0.3 ^††^	0.1 ^††^
	≤300 m	52 of 95 (54.7%)	32 of 67 (47.8%)	60 of 124 (48.4%)	144 of 286 (50.3%)	0.4 ^‡^	0.4 ^‡^	0.3 ^‡^	0.6 ^‡^
	300 < m <750	19 of 95 (20%)	13 of 67 (19.4%)	34 of 124 (27.4%)	66 of 286 (23%)				
	≥750 m	24 of 95 (25.3%)	22 of 67 (32.8%)	30 of 124 (24.2%)	76 of 286 (26.6%)				
Demographic characteristics									
Gender	Male	63 of 96 (65.6%)	32 of 67 (47.8%)	64 of 124 (51.6%)	159 of 287 (55.4%)	0.04 ^‡^	0.04 ^‡^	0.6 ^‡^	0.02 ^‡^
	Female	33 of 96 (34.4%)	35 of 67 (52.2%)	60 of 124 (48.4%)	128 of 287 (44.6%)				
**Mean age**	Years (range)	62 (16–95)	73 (32–101)	71 (21–101)	71 (16–101)	0.0001 ^†^	0.0005 ^††^	0.5 ^††^	0.0001 ^††^
Pathological characteristics									
**Localization** ^§^	Head&Neck&Face ^a^	20 of 96 (20.8%)	11 of 67 (16.4%)	38 of 124 (30.6%)	69 of 287 (24%)	0.02 ^‡^	0.02 ^‡^	0.1 ^‡^	0.08 ^‡^
	Trunk ^b^	43 of 96 (44.8%)	21 of 67 (31.3%)	33 of 124 (26.6%)	97 of 287 (33.8%)				
	Upper Limb ^c^	8 of 96 (8.3%)	13 of 67 (19.4%)	19 of 124 (15.3%)	40 of 287 (14%)				
	Lower Limb ^d^	25 of 96 (26%)	22 of 67 (32.8%)	29 of 124 (23.4%)	76 of 287 (26.5%)				
	Other sites ^e^	0 of 96 (0%)	0 of 67 (0%)	5 of 124 (4%)	5 of 287 (1.7%)				
**Stage**	I B	17 of 96 (17.7%)	2 of 67 (3%)	8 of 124 (6.5%)	27 of 287 (9.4%)	0.005 ^‡^	0.005 ^‡^	0.2 ^‡^	0.04 ^‡^
	II	51 of 96 (53.1%)	43 of 67 (64.2%)	88 of 124 (71%)	182 of 287 (63.4%)				
	III	23 of 96 (24%)	15 of 67 (22.4%)	15 of 124 (12.1%)	53 of 287 (18.5%)				
	IV	5 of 96 (5.2%)	3 of 67 (4.5%)	7 of 124 (5.6%)	15 of 287 (5.2%)				
	unknown	0 of 96 (0%)	4 of 67 (6%)	6 of 124 (4.8%)	10 of 287 (3.5%)				
**Lymph nodes**	Positive	25 of 96 (26%)	14 of 67 (20.9%)	16 of 124 (12.9%)	55 of 287 (19.2%)	0.07 ^‡^	0.02 ^‡^	0.1 ^‡^	0.5 ^‡^
	Negative	71 of 96 (74%)	50 of 67 (74.6%)	101 of 124 (81.5%)	222 of 287 (77.4%)				
	unknown	0 of 96 (0%)	3 of 67 (4.5%)	7 of 124 (5.6%)	10 of 287 (3.5%)				
**Breslow’s thickness**	Median in mm (range)	3.2 (1.8−15)	3.6 (1.9−30)	3.5 (1.8−22)	4 (1.8−30)	0.1 ^†^	0.3^††^	0.2^††^	0.07 ^††^
Ulceration status	yes	45 of 96 (47%)	31 of 67 (46.3%)	57 of 124 (46%)	133 of 287 (46.3%)	0.9 ^‡^	0.7 ^‡^	0.9 ^‡^	0.8 ^‡^
	no	49 of 96 (51%)	32 of 67 (47.8%)	57 of 124 (46%)	138 of 287 (48.1%)				
	unknown	2 of 96 (2%)	4 of 67 (6%)	10 of 124 (8%)	16 of 287 (5.6%)				
Histotype ^¶^	NOS ^f^	28 of 96 (29.2%)	16 of 67 (23.9%)	31 of 124 (25%)	75 of 287 (26.1%)	0.1 ^‡^	0.08 ^‡^	0.2 ^‡^	0.7 ^‡^
	Nodular ^g^	47 of 96 (49%)	33 of 67 (49.3%)	56 of 124 (45.2%)	136 of 287 (47.5%)				
	Superficial spreading ^h^	14 of 96 (14.6%)	12 of 67 (17.9%)	13 of 124 (10.5%)	39 of 287 (13.6%)				
	Acral ^i^	0 of 96 (0%)	1 of 67 (1.5%)	6 of 124 (4.8%)	7 of 287 (2.4%)				
	Others ^l, m, n, o, *p*, q, r^	7 of 96 (7.3%)	4 of 67 (6%)	18 of 124 (14.5%)	29 of 287 (10.1%)				
	Unknown	0 of 96 (0%)	1 of 67 (1.5%)	0 of 124 (0%)	1 of 287 (0.3%)				

**^†^** Ordinary one-way ANOVA test (the unknown cases were excluded from the analysis). ^††^ Unpaired *t*-test (the unknown cases were excluded from the analysis). ^‡^ Chi-square test (the unknown cases were excluded from the analysis). Underlined *p* highlights statistically significant results by univariate analysis (*p* < 0.05). ^§^ ICD-O code was used to define anatomical tumor localization: ^a^. C44.0; C44.1; C44.2; C44.3; C44.4; ^b^. C44.5; ^c^. C44.6; ^d^. C44.7 and ^e^. C51.0. ^¶^ SNOMED code was used to define tumor histotypes: ^f^. not specified, ^g^. M87213; ^h^. M87433; ^i^. M87443; ^l^. M87453; ^m^. M87723; ^n^. M87733; ^o^. M87803; *^p^*. M87303; ^q^. M87423; ^r^. M87713.

**Table 4:** Results of the Cox multivariate analysis (*p* < 0.0001).

Variable		Haz. Ratio	*p* > *z*	95% Conf. Interval
Age at diagnosis		1.02	0.02 ^1^	1.00−1.04
		*1.01* ^2^	*0.2*	*0.99−1.04*
Gender		0.62	0.1	0.33−1.14
		*0.65*	*0.2*	*0.32−1.33*
Altitude of Residence		1.00	0.004	1.00−1.00
		*1.00*	*0.03*	*1.00−1.00*
Anatomical Site		1.13	0.3	0.90−1.40
		*1.01*	*0.9*	*0.77−1.32*
Geographic area		1.13	0.5	0.69−1.87
		*1.13*	*0.8*	*0.42−3.01*
*TRPM1*		0.76	0.5	0.36−1.61
		*0.72*	*0.5*	*0.29−1.80*
*TYRP1*		2.43	0.01	1.24−4.77
		*2.51*	*0.02*	*1.15−5.48*
*PDL1 (CD274)*		0.71	0.3	0.37−1.35
		*0.49*	*0.06*	*0.23−1.01*
*CD2*		0.75	0.4	0.39−1.42
		*0.94*	*0.9*	*0.46−1.93*
*MITF*		0.51	0.04	0.27−0.97
		*0.98*	*0.9*	*0.44−2.19*
*HIF1A*		1.15	0.6	0.66−2.00
		*1.39*	*0.4*	*0.69−2.75*
*miR-150-5p*		0.96	0.9	0.48−1.93
		*0.62*	*0.3*	*0.27−1.42*
*miR-155-5p*		0.95	0.9	0.50−1.79
		*0.86*	*0.7*	*0.41−1.80*
*miR-204-5p*		0.87	0.7	0.48−1.59
		*1.08*	*0.8*	*0.53−2.19*
*miR-211-5p*		0.99	1	0.44−2.25
		*0.58*	*0.3*	*0.21−1.60*
Mutational Status		1.98	<0.001	1.40−2.25
		*1.55*	*0.04*	*1.01−2.39*
Stage at diagnosis	II	0.67	0.4	0.25−1.81
		*0.95*	*0.9*	*0.27−3.31*
	III	1.38	0.5	0.49−3.94
		*1.87*	*0.4*	*0.48−7.32*
	IV	9.89	<0.001	2.76−35.5
		*11.1*	*0.008*	*1.88−65.8*
Ulceration		1.3	0.4	0.72−2.34
		*1.5*	*0.3*	*0.73−2.99*
phtest *p* = 0.3				
*phtest p = 0.1*				

^1^: Underlined *p* highlights statistical significance; ^2^: In italics are reported the values of the Cox regression by exclusion of patients from Innsbruck and its land.

## Supplementary Materials

The following are available online at https://www.mdpi.com/2072-6694/12/10/2796/s1, Figure S1: Box plot representing patients’ age between genders, Figure S2: Box plot representing mRNA expression levels resulted different according to patients’ age, Figure S3: Box plot representing mRNA and miRNA expression levels resulted different according to the anatomical site, Figure S4: Box plot representing mRNA and miRNA expression levels resulted different according to the presence or absence of lymph nodes, Figure S5: Box plot representing mRNA and miRNA expression levels resulted different according to the presence of BRAF and NRAS mutations title, Figure S6: Bar plot representing the percentage of CM cases per tumor stage and lymph node involvement divided by molecular subtype, Figure S7: Kaplan Meier survival curves for overall survival (OS) adjusted for age at diagnosis for altitude of residence ranges, Figure S8: Kaplan Meier survival curves for relapse free survival (RFS) for CD2, Table S1: Primers and TaqMan probes for gene expression analysis, Table S2: Sequences and accession numbers of microRNAs, Table S3: Results of standard curves to set up qPCR analyses, Table S4: Results of the Cox Multivariate Analysis (*p* < 0.0001) for overall survival in the entire cohort of patients, Table S5: Results of associations of HIF1A expression levels with environmental, clinical and pathological variables, Table S6: Results of correlation of HIF1A expression levels with miR-155-5p.The supplementary Materials include also explanations.
