# Supplementary material for: The Association of Residential Altitude on the Molecular Profile and Survival of Melanoma: Results of an Interreg Study
Source: Cancers (Basel). 2020 Sep 29;12(10):2796. doi: 10.3390/cancers12102796 (PMC7599639; doi:10.3390/cancers12102796)
Supplement: Supplementary file 1 [file cancers-12-02796-s001.pdf]

cancers

MDPI

Supplementary Materials: The association of residential altitude on the molecular profile and survival of melanoma: Results of an Interreg study

Materials and Methods

Table S1. Primers and TaqMan probes for gene expression analysis.

Name	NM Number	Primers and Probe Sequences		Size (bp)	Annealing/ Extension Temp.
Actin Beta (*ACTB*)	NM_001101	Forward<br>Reverse<br>Probe	5′-GTGGATCAGCAAGCAGGAGT-3′<br>5′-AGGTGTAACGCAACTAAGTC-3′<br>5′- CACCGCAAATGCTTC-3′	95bp	54 °C
Glyceraldehyde-3-phoshate dehydrogenase (*GAPDH*)	NM_002046.6	Forward<br>Reverse<br>Probe	5′-GTCAAGGCTGAGAACGGGAAG- 3′<br>5′- CAAAATCAAGTGGGGCGATG- 3′<br>5′- CCAGGAGCGAGATC- 3′	91bp	54 °C
Hydroxymethylbilane synthase (*PDGB* or *HMBS*)	NM_000190.3	Forward<br>Reverse<br>Probe	5′-GATGCTGTTGTCTTTCACCCAAA-3′<br>5′- AACCAGCTCCCTGCGAAGA- 3′<br>5′-TGCCAGAGAAGAGTGTG- 3′	90bp	56 °C
Hypoxanthine phosphoribzosyltransferase 1 (*HPRT1*)	NM_000194.2	Forward<br>Reverse<br>Probe	5′-GTGTCATTAGTGAAACTGGAAAAGCA- 3′<br>5′-CGATGTCAATAGGACTCCAGATGTT- 3′<br>5′-AGCCTAAGATGAGAGTTCAAGTTGAGTTTGG- 3′	91bp	60 °C
Homo Sapiens CD2 molecule (*CD2*)	NM_001328609.1	Forward<br>Reverse<br>Probe	5′-GTAGCCAGCTTCCTTCTGATTT-3′<br>5′-GGTTTCCAAGGCATTCGTAAT-3′<br>5′- TGTTTCTTCCAAAGGTGCAGTCTCC-3′	78bp	53 °C
CD274 molecule or Programmed cell death 1 ligand 1 (*CD274* or *PDL1*)	NM_014143.4	Forward<br>Reverse<br>Probe	5′-GCTGAATTGGTCATCCCAG- 3′<br>5′- GATGGCTCCCAGAATTACCA- 3′<br>5′- CCTCTGGCACATCCTCCAAATGA- 3′	78bp	53 °C

Transient receptor potential cation channel subfamily M member 1 (*TRPM1*)	NM_001252020.1	Forward<br>Reverse<br>Probe	5′-AGCTGCTGAACTGGGTGAAT- 3′<br>5′-TCAGGAGCTTCACAAAGTCG- 3′<br>5′-TTGGAGCAAGCGATGCTAGATGC- 3′	82bp	60 °C
Tyrosinase related protein 1 (*TYRP1*)	NM_000550.2	Forward<br>Reverse<br>Probe	5′-CACGCCTCCTTTTTATTCCA- 3′<br>5′- GTCATACTTTCCCGTGGGG- 3′<br>5′-CAGTTTCCGAAACACAGTGGAAGGTT- 3′	84bp	60 °C
Melanogenesis associated transcription factor (*MITF*)	NM_001354604.1	Forward<br>Reverse<br>Probe	5′-CAGCGTGTATTTTTCCCACA- 3′<br>5′-TTCAATCAGGTTGTGATTGTC- 3′<br>5′-AGAGCACTGGCCAAAGAGAGGCA- 3′	83bp	60 °C
Hypoxia-inducible factor 1-alpha (*HIF1A*)	AF304431.1	Forward<br>Reverse<br>Probe	5′-ATGAACATAAAGTCTGCAACATGGA- 3′<br>5′- CTGAGGTTGGTTACTGTTGGTATCA- 3′<br>5′-TTGCACTGCACAGGCCACATTCAC- 3′	84bp	58 °C

Table S2. Sequences and accession numbers of microRNAs.

Name	Accession Number	Sequence	Qiagen Cat. No.	Annealing Temp.
hsa-let-7e-5p	MIMAT0000066	5'UGAGGUAGGAGGUUGUAUAGUU	YP00205711	60 °C
hsa-miR-423-3p	MIMAT0001340	5'AGCUCGGUCUGAGGCCCCUCAGU	YP00204488	60 °C
has-miR-150-5p	MIMAT0000451	5'UCUCCCAACCCUUGUACCAGUG	YP00204660	60 °C
has-miR-155-5p	MIMAT0000646	5'UUAAUGCUAAUCGUGAUAGGGGU	YP00204308	60 °C
has-miR-204-5p	MIMAT0000265	5'UUCCCUUUGUCAUCCUAUGCCU	YP00206072	60 °C
has-miR-211-5p	MIMAT0000268	5'UUCCCUUUGUCAUCCUUCGCCU	YP00204009	60 °C

cancers MDPI

Molecular Subtypes

To define molecular subtypes, gene expression was dichotomized into high or low level depending if the expression level in term of fold change was higher or lower than its median value. Accordingly, each transcriptomic subtype was described as follows: "keratin" with high level of *MITF*, *TYRP1*, and low expression levels of *miR-150-5p*; "MITF-low" had low expression of *MITF*, *TYRP1*, and high expression of *miR-204-5p*; the "immune" subtype was characterized by high expression of *CD2*, *PDL1* (*CD274*) and *miR-150-5p*.

Results

mRNAs and microRNAs Real-Time PCR

The results of real-time PCR efficiencies for each primer set were reported in Table 3. Although efficiencies were less than 100%, the ΔΔ Ct method [23] was applied because all the regression lines were parallel and efficiencies values were comparable. As housekeeping, the geometric mean of at least two genes was employed. Based on their stability and their expression levels, the geometric mean of *HPRT1* and *PDGB* was used to normalize *CD2*, *CD274*, *HIF1A* and *TRPM1*; while *ACTB* and *GAPDH* were used for *TYRP1* and *MITF* normalization and the geometric mean of *Let-7e-5p* and *miR-423-3p* was used to normalize all the miRNAs. As calibrator was used a pool of cDNA made of 10 samples from the cohorts.

Table S3. Results of standard curves.

Target	Slope	Intercept	Efficiency (%)
ACTB	-3.5	28.5	93
GAPDH	-3.5	29	93
PDGB or HMBS	-3.7	35	84
HPRT1	-3.9	34	80.5
CD2	-3.8	30	83
CD274 (or PDL1)	-4	38	75
TRPM1	-4.12	36	75
TYRP1	-3.8	30	83
MITF	-3.9	33.5	80
HIF-1a	-3.5	33.9	92
Let-7e-5p	-3.6	25.7	88
miR-423-3p	-3.3	25.8	100
miR-150-5p	-3.6	26.2	88
miR-155-5p	-3.5	26.7	92
miR-204-5p	-3.9	27	80
miR-211-5p	-3.5	25	95

cancers MDPI

Patients Features

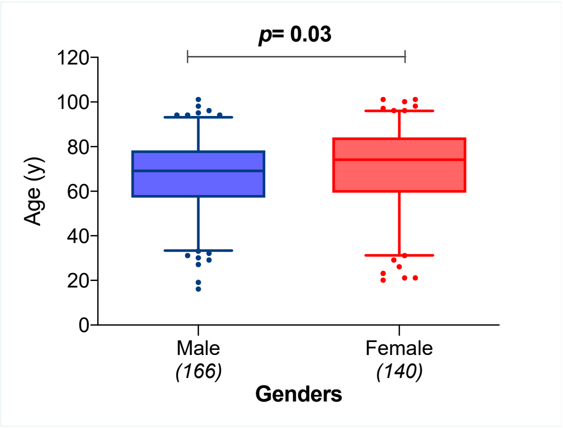

Figure S1. Box plots representing patients' age between genders. The box extends from the 25th to the 75th percentiles, the middle line indicates the median and the whiskers are the 5th and 95th percentiles. Female results elder than male patients (p = 0.03; Mann-Whitney test).

Transcriptomic Profile and Demography

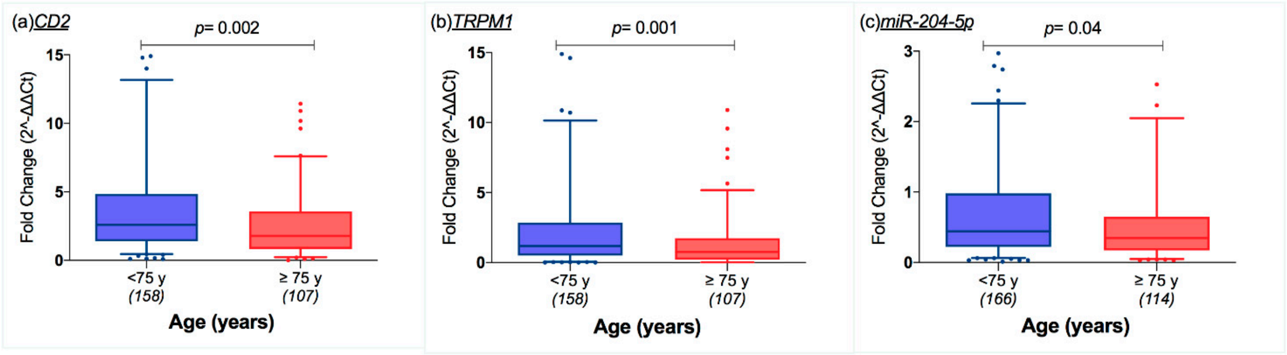

Figure S2. Box plots representing mRNA expression levels (ratio of threshold cycles) resulted different according to patients' age: (a) CD2, (b) TRPM1 and (c) miR-204-5p were less expressed in patients over 75 years old (p = 0.002, p = 0.001 and p = 0.04, in order). The box extends from the 25th to the 75th percentiles, the middle line indicates the median and the whiskers are the 5th and 95th percentiles (Mann-Whitney test).

Transcriptomic Profile and CM Features

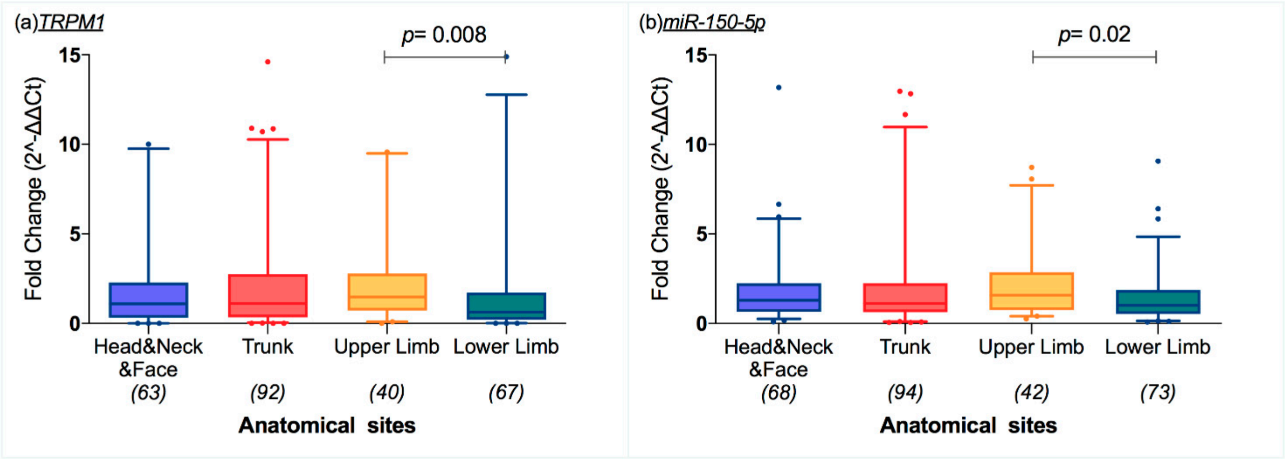

Figure S3. Box plots representing mRNA and miRNA expression levels (ratio of threshold cycles) resulted different according to the anatomical sites: CM located in upper limb showed higher expression of (a) TRPM1 and (b) miR-150-5p than CM located in lower limb ($p = 0.008$ and $p = 0.02$, respectively). The box extends from the 25th to the 75th percentiles, the middle line indicates the median and the whiskers are the 5th and 95th percentiles (Mann-Whitney test).

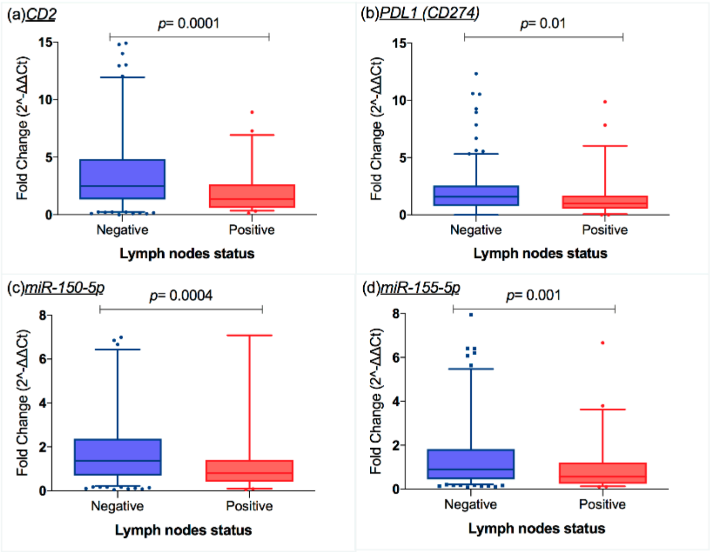

Figure S4. Box plots representing mRNA and miRNA expression levels (ratio of threshold cycles) resulted different according to the presence or absence of lymph nodes: (a) CD2, (b) PDL1, (c) miR-150-5p and (d) miR-155-5p were less expressed in patients with positive lymph nodes ($p = 0.0001$, $p = 0.01$, $p = 0.0004$ and $p = 0.001$, in order). The box extends from the 25th to the 75th percentiles, the middle line indicates the median and the whiskers are the 5th and 95th percentiles (Mann-Whitney test).

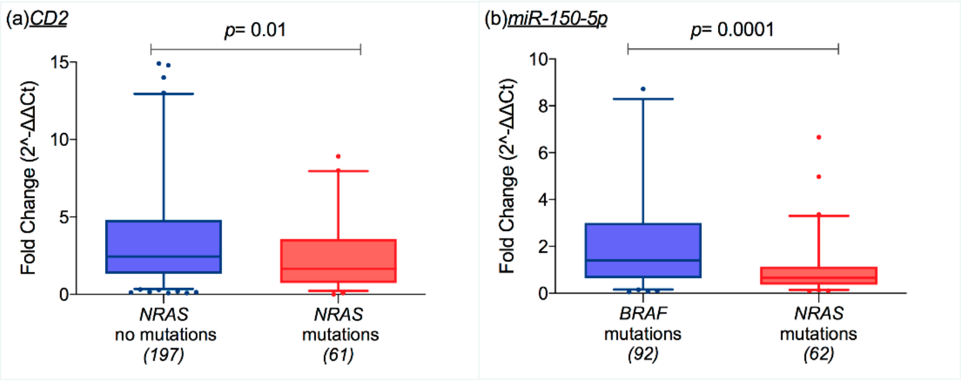

Figure S5. Box plots representing mRNA and miRNA expression levels (ratio of threshold cycles) resulted different according to the presence of BRAF and NRAS mutations. (a) CD2 was more expressed in wild type NRAS patients than RAS mutated ones (p = 0.01). (b) miR-150-5p was more expressed in patients with BRAF mutation than those with RAS mutations (p = 0.0001). The box extends from the 25th to the 75th percentiles, the middle line indicates the median and the whiskers are the 5th and 95th percentiles (Mann-Whitney test).

Molecular Subtypes

According to the transcriptomic classification as defined in the material and method section CM were grouped as follows: 52 CM resulted of "keratin" type, 37 "MITF-low", 58 "immune" and 117 as unclassified.

Among the tumor characteristics, the distribution of CM stages across molecular subtypes resulted significant different, namely the defined "immune" cluster was more represented in stage I and II, while the "keratin" group appeared more numerous in stage III (p = 0.009, Fig. 6a). Furthermore, in our dataset samples with negative lymph nodes belonged mostly to "immune" cluster, on contrary the "keratin" one fitted in positive lymph nodes samples (p = 0.0006; Supplementary Figure 6b).

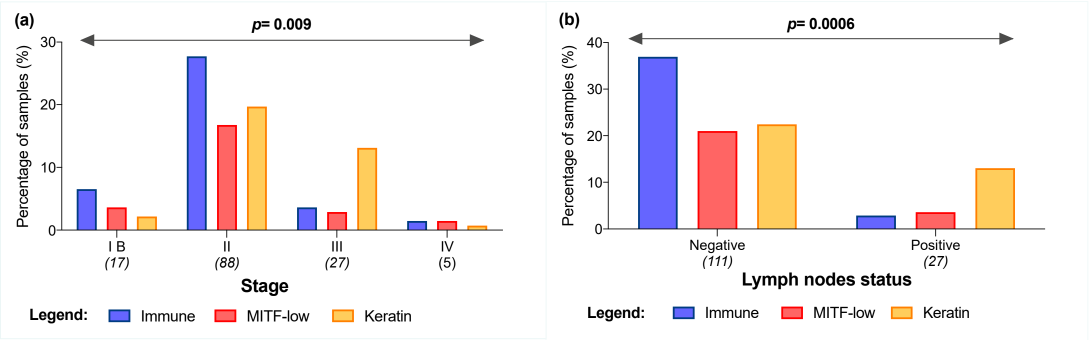

Figure S6. (a) Bar plots representing the percentage of CM cases per tumor stage divided by molecular subtype, unclassified CM were excluded from the analysis (p value refers to extended Wilcoxon rank-sum test). The "immune" cluster was more represented in stage I and II, while the "keratin" one was more numerous in stage III (p = 0.009). **(b)** Bar plot representing the percentage of CM cases per lymph nodes positivity divided by molecular subtypes; unclassified CM were excluded from the analysis (p value refers to Chi-square test). Negative lymph nodes samples belong mostly to "immune" group, while the positive lymph nodes patients belong to "keratin" cluster (p = 0.0006).

Overall Survival

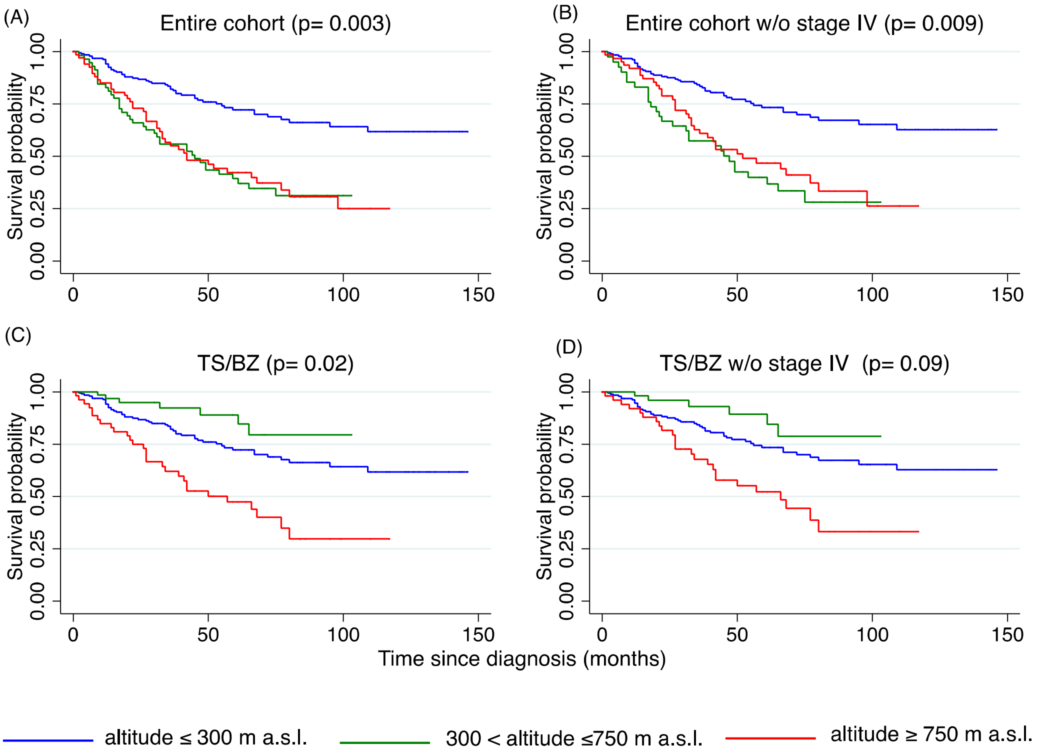

Figure S7. Kaplan Meier survival curves for overall survival (OS) adjusted for age at diagnosis for altitude of residence ranges (under 300 meters, between 300 and 750 meters and above 750 m a.s.l.; (a), entire cohort, p = 0.003; (b), entire cohort without stage IV, p = 0.009; (c) in cases from TS/BZ, p= 0.02; (d) in cases from TS/BZ without stage IV CM, p = 0.009. The graphs of the survivor function were adjusted to the mean age of patients. OS: Overall survival (p values on the graph refer to the log-rank test).

The Cox proportional hazard regression method was applied to analyze patients' demographics (age at diagnosis, gender, altitude of residence, geographical areas), pathological covariates (stage, ulceration, anatomical site), mutational status, gene and miRNA expression levels in the entire cohort of patients, to test the joint effects of the covariates on patients' overall survival. The same analysis excluding patients from Innsbruck and its land did not fit the proportional hazard assumption, therefore results were not reported.

Table S4. Results of the Cox Multivariate Analysis (p < 0.0001) for overall survival in the entire cohort of patients.

Variable	Haz. Ratio	$p > z$	95% Conf. Interval
Age at diagnosis	1.04	<0.001 [1]	1.03-1.06
Gender	0.81	0.4	0.51-1.28
Altitude of Residence	1.00	0.05	1.00-1.00
Anatomical Site	1.04	0.7	0.87-1.25
Geographic area	1.21	0.3	0.83-1.77
TRPM1	0.71	0.3	0.40-1.28
TYRP1	2.08	0.005	1.24-3.49
PDL1 (CD274)	0.84	0.5	0.51-1.37
CD2	0.56	0.03	0.34-0.94
MITF	0.61	0.06	0.37-1.01

HIF1A		0.81	0.3	0.52-1.25
miR-150-5p		0.86	0.5	0.52-1.41
miR-155-5p		1.08	0.8	0.66-1.77
miR-204-5p		1.17	0.5	0.74-1.85
miR-211-5p		1.40	0.3	0.74-2.66
Mutational Status		1.50	0.003	1.15-1.97
Stage at diagnosis	II	1.12	0.8	0.48-2.64
	III	1.25	0.6	0.49-3.19
	IV	6.05	0.002	1.96-18.8
Ulceration		1.37	0.2	0.86-2.17
phtest $p = 0.1$				

1: Underlined p highlights statistical significance.

Relapse-free Survival

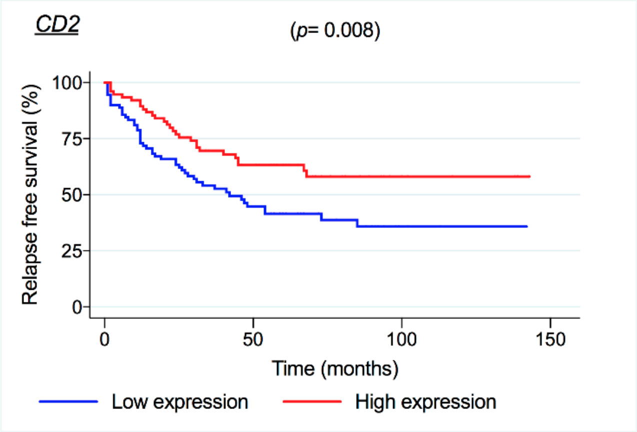

Figure S8. Kaplan Meier survival curves for relapse free survival (RFS) for CD2 (p = 0.008). CD2 mRNA expression was dichotomized in low expression and high expression for the median value of each transcript (p values on the graph refers to the Log-rank test).

Analysis of HIF1A

The expression level of HIF1A was analyzed in the entire cohort of patients. Results are reported in the Table S5.

Table S5. Results of associations of HIF1A expression levels with environmental, clinical and pathological variables.

**Variable**	***p* value**	**Variable**	***p* value**
Gender	0.7	Anatomical site	0.5
Geographic Area	0.4	Ulceration	0.6
Altitude range	0.5	Lymph node involvement	0.2
Melanoma Stage	0.3	Mutational status	0.5

The influence of HIF1A mRNA on patients' survival was investigated by dichotomizing HIF1A expression levels according to its median value. Over the entire cohort, HIF1A did not result to influence on melanoma specific survival ($p = 0.4$); overall survival ($p = 0.06$) and relapse free survival (0.1).

To explore on the regulatory effect of miR155-5p on HIF1A transcript a correlation analysis was carried out comparing ΔΔCt ratio of miR155-5p with those of HIF1A in the entire cohort or samples as well as grouping them in the different altitude ranges. Results are listed in Table S6.

Table S6. Results of correlation of HIF1A expression levels with miR-155-5p.

p Value	R^2	Group
0.9	*<0.0001*	Entire cohort
0.9	*0.0002*	Altitude ≤ 300 m a.s.l.
0.7	*0.0026*	300 < altitude ≤ 750 m a.s.l.
0.6	*0.005*	Altitude > 750 m

© 2020 by the authors. Licensee MDPI, Basel, Switzerland. This article is an open access article distributed under the terms and conditions of the Creative Commons Attribution (CC BY) license (http://creativecommons.org/licenses/by/4.0/).
